# CoQ Regulates Brown Adipose Tissue Respiration and Uncoupling Protein 1 Expression

**DOI:** 10.3390/antiox12010014

**Published:** 2022-12-22

**Authors:** Ching-Fang Chang, Amanda L. Gunawan, Irene Liparulo, Peter-James H. Zushin, Ambre M. Bertholet, Yuriy Kirichok, Andreas Stahl

**Affiliations:** 1 Department of Nutritional Sciences and Toxicology, University of California, Berkeley, CA 94720, USA; 2 Department of Physiology, University of California, San Francisco, San Francisco, CA 94158, USA

**Keywords:** Coenzyme Q, brown adipose tissue, mitochondrial function, thermogenesis

## Abstract

Coenzyme Q (CoQ, aka ubiquinone) is a key component of the mitochondrial electron transport chain (ETC) and membrane-incorporated antioxidant. CoQ10 deficiencies encompass a heterogeneous spectrum of clinical phenotypes and can be caused by hereditary mutations in the biosynthesis pathway or result from pharmacological interventions such as HMG-CoA Reductase inhibitors, and statins, which are widely used to treat hypercholesterolemia and prevent cardiovascular disease. How CoQ deficiency affects individual tissues and cell types, particularly mitochondrial-rich ones such as brown adipose tissue (BAT), has remained poorly understood. Here we show that pharmacological and genetic models of BAT CoQ deficiency show altered respiration that can only in part be explained by classical roles of CoQ in the respiration chain. Instead, we found that CoQ strongly impacts brown and beige adipocyte respiration via the regulation of uncoupling protein 1 (UCP1) expression. CoQ deficiency in BAT robustly decreases UCP1 protein levels and uncoupled respiration unexpectedly, resulting in increased inner mitochondrial membrane potential and decreased ADP/ATP ratios. Suppressed UCP1 expression was also observed in a BAT-specific in vivo model of CoQ deficiency and resulted in enhanced cold sensitivity. These findings demonstrate an as yet unappreciated role of CoQ in the transcriptional regulation of key thermogenic genes and functions.

## 1. Introduction

Coenzyme Q (CoQ) is widely known as one of the components of the oxidative phosphorylation (OXPHOS) chain in the mitochondria, responsible for carrying electrons from multiple entry points to Complex III. While the functions of CoQ in the mitochondrial electron transport chain are well explored, basic questions about CoQ physiology, such as its transport [1], participation in redox reactions outside of mitochondria [2], and roles in mitochondrial to nuclear communications are still under investigation.

Human CoQ deficiencies, caused by either genetic or pharmacological inhibition of CoQ synthesis, have a plethora of phenotypes, and clinical consequences are highly variable. Different impacts on multiple organs, and the underlying molecular mechanisms, still represent a challenging question to be answered. Primary CoQ deficiency is usually associated with multisystem involvement, and the major phenotypes are predominantly encephalomyopathy, severe infantile multisystemic disease, nephropathy, cerebellar ataxia, and isolated myopathy [3,4]. Secondary CoQ deficiency, occurring in conditions not directly linked to mutations in the COQ or PDSS genes, also comprises a broader spectrum of disorders frequently associated with drug treatments, such as HMG-CoA reductase inhibitors, ‘statins’ and Amitriptyline therapy. Further, serum and tissue CoQ10 concentrations in individuals with metabolic disorders, such as diabetes, cardiovascular diseases, or hypercholesterolemia, are also decreased due to incompletely understood reasons resulting in altered lipid and redox homeostasis [5,6]. While the clinical benefits of CoQ supplementation remain controversial [7], it is clear that statins have the ability to lower serum levels of CoQ [8] and are associated with mitochondrial dysfunction in muscle [9]. CoQ deficiency commonly affects organs rich in mitochondria and with high energy demand, such as the heart, brain, and kidney [10].

Brown adipose tissue (BAT) facilitates nonshivering thermogenesis through the actions of uncoupling protein 1 (UCP1), which allows for the dissipation of metabolic energy in the form of heat. It features highly active and densely packed mitochondria [11,12]. In the last two decades, BAT has received considerable interest for its multifaceted roles in adult human metabolism [13], and it has also been described as a potential target for the treatment of a broad range of metabolic disorders, such as obesity. How CoQ deficiency affects BAT has not been well studied. Thus far, muscle biopsies are the most common clinical method to detect primary CoQ deficiency [14], and CoQ levels in adipose tissues of primary CoQ deficiency patients have not been reported to our knowledge. Despite this, Raman et al. reported a clinical case of a neonate with ubiquinone deficiency presenting hypothermia at 6 h of age [15].

Previously, we showed that BAT is a major sink for circulating CoQ and that its uptake is dependent on the scavenger receptor CD36 [1]. In this paper, we created pharmacological and genetic BAT CoQ deficiency models by inhibiting endogenous synthesis. This approach uncovered a novel role of CoQ in the transcriptional regulation of UCP1 and, thus, respiratory and thermogenic function.

## 2. Materials and Methods

### 2.1. Mouse Model

All animal experiments were approved and performed under the guidelines and ethical regulations established by the UC Berkeley Animal Care and Use Committee. Brown fat-specific PDSS2 knockout PDSS2^BKO^ were generated by crossing PDSS2-LoxP mice (gift from Dr. Gasser) [16] with UCP1-cre mice (mouse model developed by the Rosen lab deposited in JAX, stock #024670) on a C57BL/6J background. The animals were fed a regular diet (LabDiet 5053) under the ambient temperature of 23 °C. Male animals aged between 6-8-week-old were fed a defined low CoQ diet (Research diet D12450J) for two to three weeks before experiments unless described otherwise. The defined low CoQ diet contains only 32% of CoQ content compared to normal chow and thus is suitable in our experiments for limiting CoQ intake. PDSS2 flox allele control animals (PDSS2^FL^) were used as control relative to PDSS2^BKO^. All animal studies were performed using age-matched male mice (6–12 weeks) and repeated at least twice.

### 2.2. Lentiviruses

Doxycycline inducible lentiviral Cre recombinase expression plasmid pLenti-Cre was constructed by replacing the complete attR1-CmR-ccdB-attR2 of the destination vector pInducer20, a gift from Stephen Elledge [17] (Addgene plasmid # 44012; RRID:Addgene_44012), with the Cre recombinase gene of the entry vector pENTR1a-cre (a gift from Xin Chen at UCSF) using Gateway cloning technique (Invitrogen, Waltham, MA, USA). Lentiviruses were produced using Lipofectamine3000 (Invitrogen) according to the manufacturer’s instructions. Briefly, 293T cells were transfected with pLenti-cre, with psPAX2 (a gift from Didier Trono, Addgene plasmid # 12260; RRID: Addgene_12260), pMD2.G (gift from Didier Trono, Addgene plasmid # 12259; RRID: Addgene_12259) in Lipofectamine3000. Supernatants were harvested at 24 h and 48 h later, clarified, filtered through a 0.45-μm filter, and stored at −80 °C.

### 2.3. Cell Cultures

Murine brown/beige cells: Immortalized murine brown preadipocyte line is a generous gift from Kajimura Lab [18]. The SVFs were isolated from BAT fat depots of either C57/B6 or PDSS2^FL^ male mice aged 8-12 weeks. Cells were cultured in Dulbecco’s Modified Eagle Medium (DMEM) supplemented with 10% fetal bovine serum (FBS), 5% penicillin, and streptomycin (P/S). Murine brown adipocytes were prepared from adipogenic differentiation of the confluent preadipocytes/SVFs with DMEM containing 10% FBS, penicillin/streptomycin, and differentiation cocktails (850 nM insulin, 2 nM T3, 125 nM indomethacin, 0.5 mM 3-isobutyl-1-methylxanthine (IBMX), 1µM dexamethasone, and 1 µM rosiglitazone) for two days. Two days after induction, cells were switched to a maintenance medium containing 10%FBS, P/S, insulin, T3, and rosiglitazone. Experiments assessing CoQ deficiency on thermogenic function were performed on day 4–6 of differentiation (48 h) or day 5–6 (24 h) in the presence of 4-chlorobenzoic acid (4CBA, 4 mM) or vehicle (0.1% DMSO and 1% ethanol in culture medium) for murine adipocytes. Primary beige adipocytes were treated at days six to eight, based on when the expression of adipocyte markers fatty acid-binding protein 4 (FABP4) and UCP1 reached a plateau (indicators of adipocyte maturation), n = 3 independent biological replicates per condition. Antioxidants were added with 4CBA (4 mM) in the maintenance medium at day five of differentiated adipocytes for 24 h for assessment of UCP1 rescue effects. N-Acetyl Cysteine (NAC) (250 μM), mitoquinone (mitoQ) (2 μM), MitoTEMPOL (2 μM), vitamin E antioxidants alpha-tocopherol phosphate disodium salt (vitE(TP)) (10 μM), alpha-tocopheryl quinone (VitE(TQ)) (10 μM).

Human beige cells: Human mesenchymal stem cells isolated from subcutaneous fats were purchased from ZenBio and cultured in DMEM/F12 supplemented with 10% FBS, 5 ng/mL bFGF, and FGF, 10 mM N-2-hydroxyethylpiperazine-N-2-ethane sulfonic acid (HEPES), penicillin, and streptomycin. Beige adipogenesis was induced two days post-confluent with DMEM/F12 (10%FBS, 10 mM HEPES, P/S) in the presence of the aforementioned differentiation cocktail for three days (Days 0–3). Cells were then switched to DMEM/F12 (10%FBS, 10 mM HEPES, Pen/Strep) containing insulin and T3 for five days. 4CBA or vehicle was added from day eight to day ten of differentiation.

Murine white cells: 3T3L1 preadipocytes were kept in DMEM supplemented with 10% FBS, 2 mM L-glutamine, 0.25 U/mL amphotericin B, an antibiotic mixture (100 U/mL penicillin + 100 μg/mL streptomycin) until confluence. To initiate differentiation, cells were treated with DMEM containing 10% FBS, penicillin/streptomycin, and differentiation cocktails (0.5 mM IBMX, 1 μM dexamethasone, 10 μg/mL insulin) for three days. After three days cells were replaced with DMEM containing 10% FBS, penicillin/streptomycin, and 10 μg/mL insulin. After three more days, cells were returned to media containing just DMEM containing 10% FBS and penicillin/streptomycin. Cells were treated with 4CBA (4 mM) for 24 h on days 6–7 of differentiation.

Lentivirus infection: Isolated SVFs from BAT of PDSS2^FL^ male mice were cultured in DMEM supplemented with 10% FBS, penicillin, and streptomycin. Cells were then incubated with a medium containing lentivirus (pLenti-cre construct) for 16–18 h. Adipogenic differentiation was initiated once cells reached full confluency, as previously described. After five days of adipogenic differentiation, the adipocytes derived from SVFs of PDSS2^FL^ male mice were switched to a DMEM medium supplemented with doxycycline (0.5 ug/mL) and 10% lipoprotein free serum for 48 h.

### 2.4. CoQ_10_ Supplementation

MicelleQ10: CoQ10-loaded micelles were prepared as previously published [19]. Briefly, polyethylene glycol (15)-hydroxystearate (Kolliphor HS15) (30 mg) and CoQ_10_ (5 mg) were dissolved at 50 °C with constant stirring, and 3 mL of glucose solution (0.2% *w*/*v*) containing sodium chloride (0.1538 M) was added drop by drop while stirring. CoQ_10_ concentration in the micelle solution was determined by the High-Performance Liquid chromatography HPLC method. A final concentration of 2 µM of the micelle formulation CoQ_10_ was used in the described experiments.

Intralipid CoQ_10_: Intralipid CoQ_10_ was prepared as previously described [1]. Briefly, CoQ_10_ was dissolved in 100% ethanol to a concentration of 1mM and heated at 65 °C for 5 min. 10 uL of 1 mM CoQ_10_ was mixed with 90 uL of Intralipid and heated at 65 °C for 5 min. This solution was then mixed with 1 mL of prewarmed media, yielding a 10 µM final concentration of CoQ_10_, and added directly to cells.

### 2.5. Coenzyme Q Extraction and Measurement

CoQ levels from tissue cells or isolated mitochondria were extracted using hexane as previously described [1]. The extraction of CoQ from cultured cells or tissue was performed with minor modifications as described by Podda et al. [20]. Tissue or cells were homogenized in 1XPBS with 0.5 mg/mL of butylated hydroxytoluene (BHT). 0.1 mM SDS was added, and samples were vortexed and sonicated. 50 µL of the sample was removed for BCA assay protein quantification and 50 µL of 50 µM CoQ_4_ was spiked in as an internal standard to calculate extraction and recovery efficiency. 100% EtOH was added, and samples were vortexed and sonicated. Hexane was added, and samples were vortexed and centrifuged. The upper hexane layer was dried under gentle nitrogen steam in a 37 °C water bath. The dried remnants were dissolved in 100 µL 100% EtOH. Quantification of CoQ_9_ and CoQ_10_ was determined using Agilent 1100 (HPLC) system equipped with a photodiode array detector on a C18-ODS Hypersil reverse phase column (Thermo Scientific #30105-254630). The CoQ analysis was performed with a flow rate of 1 mL/min with a mobile phase consisting of solution A (80% methanol/20% water) and solution B (100% ethanol) for 40 min. CoQ_4_, CoQ_9,_ and CoQ_10_ peaks, identified at λ = 275 nm, appear around 8 min, 23.8 min, and 24.6 min, respectively. Absolute CoQ amounts were determined using a standard curve generated from commercial CoQ_9_ and CoQ_10_ (Sigma), and these amounts were normalized to protein amount.

### 2.6. Mitochondria Isolation

Mitochondria were isolated as previously described [1]. Briefly, murine brown adipocytes were trypsinized, washed in PBS, and resuspended in STE buffer (250 mM sucrose, 5 mM Tris, and 2 mM EGTA, pH 7.4) with 1% BSA. Cells were homogenized using 10 strokes with a tight fit dounce homogenizer. Cells were then centrifuged at 8500× *g* for 10 min at 4 °C and the pellet was resuspended in STE with 1% BSA. Centrifugation at 700× *g* for 10 min at 4 °C was conducted to get rid of nuclei. The nuclei pellet was discarded, and the supernatant was transferred to a new tube and centrifuged at 8500× *g* for 15 min at 4 °C to pellet the crude mitochondrial fraction.

### 2.7. Mitochondrial ROS and Mitochondrial Mass Detection

Cells were treated as described and were loaded with MitoSOX (5 μM, Molecular Probe M36008) or Nonyl Acridine Orange (NAO, 250 nM, Invitrogen A1372, Waltham, MA, USA) in culture media for 30 min at 37 °C. Single-cell suspensions were prepared using TrpLE (Invitrogen) and an LSRII (BD Biosciences, Franklin Lakes, NJ, USA) flow cytometer was used for fluorescence detection. Data were analyzed with FlowJo software.

### 2.8. ADP/ATP and NAD/NADH Measurements

Cells grew and differentiated on a 96-well plate and were treated with 4CBA (4 mM) for 48 h from day four to day six. Cells were then washed with PBS, lysed in Nucleotide Releasing Buffer, and immediately used for measuring ADP/ATP ratio with an ADP/ATP ratio assay kit (ab65313, Abcam, Cambridge, UK). For NAD/NADH ratio, cells were homogenized in NADH/NAD Extraction Buffer and centrifuged at 13,000× *g* for 10 min at 4 °C. The supernatants were subjected to NAD and NADH measurements using NAD/NADH Quantification Kit (MAK037, Sigma-Aldrich, Burlington, NJ, USA).

### 2.9. RNA Preparation and Quantitative RT-PCR

mRNA was extracted from tissues or in vitro cultures with TRIzol reagent (Invitrogen, Waltham, MA, USA) and purified using Direct-zol RNA miniprep (R2055, Zymo, Irvine, CA, USA) following manufacture instructions with DNase treatment. cDNA was synthesized using a Maxima First Strand cDNA synthesis kit (K1672, Thermo Scientific, Waltham, MA, USA), and 10–20 ng cDNA was used for qPCR on a QuantStudio 5 real-time PCR system with TaqMan Universal Master Mix II and validated PrimeTime primer-probe sets (Integrated DNA Technologies, Coralville, IA, USA). The ΔΔCt method was used to comparatively assess gene expression. Primer sequences are listed in Table A1.

### 2.10. In Vivo Respirometry

Between six to eight week old male animals were fed a defined low CoQ diet (D12450J Research Diets, New Brunswick, USA) under ambient temperature (23 °C) for two weeks. Food intake and body weight were monitored three times a week. Whole-body energy expenditure (VO_2_, VCO_2,_ and real-time) was recorded at indicated environmental temperatures using a Comprehensive Lab Animal Monitoring System (CLAMS, Columbus Instruments, Columbus, OH, USA). Data were imported and analyzed using the web-based analysis tool for indirect calorimetry experiments (CalR) [21].

### 2.11. H&E Staining and Imaging

Tissue samples were harvested from male C57/BL mice fed with a defined low CoQ diet for 21 days and were fixed in 10% formalin, embedded in parafilm, and sectioned. BAT, iWAT, eWAT, and liver were stained with hematoxylin and eosin (H&E) and were imaged using the EVOS M5000 imaging system. Images are representative of three mice per group, with three images taken per mouse.

### 2.12. In Vitro Respirometry

Oxygen consumption rate (OCR) was measured in differentiated white and brown adipocytes using the Seahorse XFe24 Extracellular Flux Analyzer (Agilent, Santa Clara, CA, USA). Cells were treated with 4CBA (4 mM) for 24 h and preincubated in the XF assay medium supplemented with 10 mM glucose, 2 mM sodium pyruvate, and 2 mM GlutaMAX at CO_2_ free incubator for an hour. Cells were subjected to the mitochondrial stress test by sequentially adding oligomycin (1 µM), FCCP (1 µM), and antimycin/rotenone mix (1 µM/1 µM). For OCR measured in UCP1 activating conditions cells were pretreated with 2% BSA and 2 µM of isoproterenol for 1 h before analysis using the mitochondrial stress test.

### 2.13. Immunoblot

BAT mitochondria were isolated as previously described [1], and protein lysates were extracted using RIPA lysis buffer (50 mM Tris pH 8.0, 1% Triton-X-100, 150 mM NaCl, 0.5% Sodium Deoxycholate, 0.2% SDS). Samples were mixed with SDS Protein Loading Buffer (Laemmli buffer) and boiled for 5 min at 95 °C. 15 ug of mitochondrial proteins or 25 ug of total cellular proteins were separated by gel electrophoresis on either a 4–20% gradient TGX gels and transferred onto PVDF or nitrocellulose membranes using Trans-Blot Turbo Transfer system (BioRad, Hercules, CA, USA). After transfer, membranes were blocked using 5% milk in TBST buffer and incubated with primary antibody overnight at 4 °C in blocking buffer. Mitochondrial electron transport chain proteins were detected using Total OXPHOS Rodent WB Antibody Cocktail (ab110413). Membranes were washed three times with TBST buffer before incubation in IRDye 680LT Goat anti-Mouse IgG antibody or IRDye 800CW Goat anti-Rabbit IgG antibody (LI-COR Biosciences) for 1 h at room temperature in blocking buffer. The membranes were washed three times with TBST, and proteins were detected using Odyssey Imaging System. Image Studio Lite was used for protein quantification. If more than one protein was probed on a membrane, the primary and secondary antibody incubation steps were repeated individually for each protein prior to imaging. Primary and secondary antibodies are listed in Table A2.

### 2.14. Lipid Peroxidation

Lipid peroxidation products malondialdehyde (MDA) was measured using TBARS assay (KGE013, R&D). BAT tissues (20–25 mg) were homogenized in RIPA buffer containing Halt protease and phosphatase inhibitors (Thermo Scientific, Waltham, USA) and centrifuged at 10,000× *g* for 5 min at 4 °C. The tissue homogenates were subjected to TBARS assay following the manufacturer’s instructions.

### 2.15. UCP1 Current Measurement

Mitoplast isolation and UCP1 current measurement were carried out as previously described [22]. Briefly, mitoplasts were isolated from BAT of C57/BL mice fed with a defined low CoQ diet for 10–15 days. Patch pipettes were filled with 130 mM tetramethylammonium hydroxide (TMA), 1 mM EGTA, 2 mM magnesium chloride, 150 mM HEPES, (pH adjusted to 7.0 with D-gluconic acid, tonicity adjusted to ∼360 mmol/kg with sucrose). Whole-mitoplast UCP1 current was recorded in the bath solution containing 150 mM HEPES and 1 mM EGTA (pH adjusted to 7 with Trizma base, tonicity adjusted to ∼300 mmol/kg with sucrose). All experiments were performed under continuous perfusion of the bath solution. Data acquisition and analysis were performed using PClamp 10 (Molecular Devices, San Jose, CA, USA) and Origin 7.5 (OriginLab, Northampton, PA, USA). All electrophysiological data presented were acquired at 10 kHz and filtered at 1 kHz. Amplitudes of H+ currents were measured 25 ms after the application of the −160 mV voltage step.

### 2.16. Statistical Analysis

All measurements were taken from distinct samples. Data were analyzed using Prism and are expressed as mean ± SEM. Statistical significance was determined by unpaired two-tailed student *t*-test or one-way ANOVA for the comparison of two conditions. Significance presented at * *p* < 0.05, ** *p* < 0.01, and *** *p* < 0.001 compared to controls or otherwise indicated.

## 3. Results

### 3.1. CoQ Regulates UCP1 Expression in BAT

**Figure 1:**
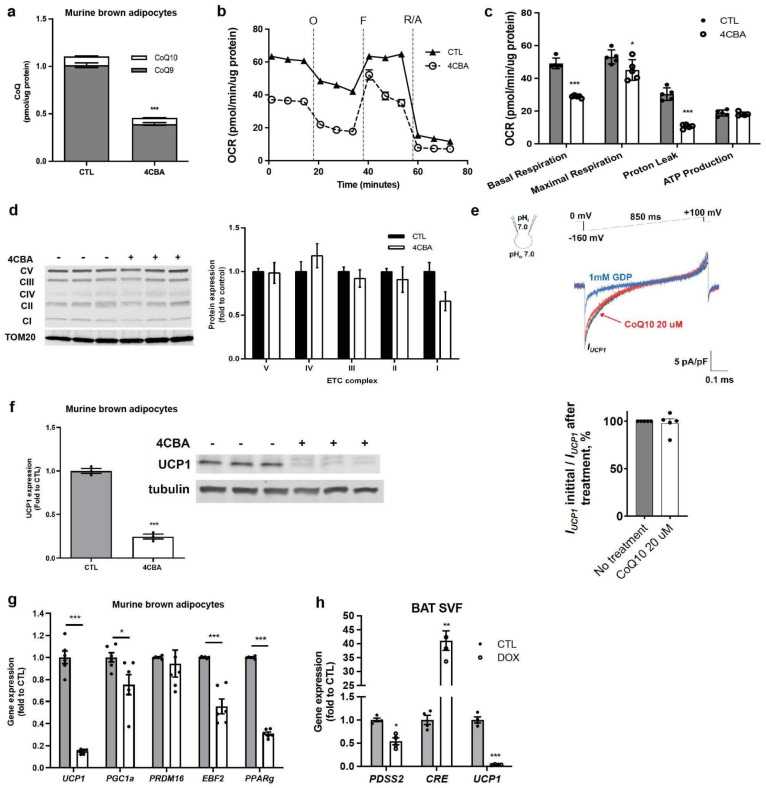
CoQ deficiency affects BAT mitochondrial respiration and UCP1 level. (**a**) Coenzyme Q (CoQ) levels in murine brown adipocytes were decreased after treatment with 4-chlorobenzoic acid (4CBA) compared to vehicle (CTL), n = 3. (**b**,**c**) Oxygen consumption rate (OCR) was measured in control, and 4CBA treated cells, using seahorse XFe24 analyzer and components of mitochondrial respiration were analyzed, n = 5. Basal, maximal, and proton leak OCR were decreased in 4CBA-treated cells compared to CTL. (**d**) Mitochondrial complexes isolated from brown adipocytes were measured by western blot, n = 3. Expression was unchanged between CTL and 4CBA-treated cells. Each lane in the western blot pictured is one independent sample. (**e**) Upper panel: Representative UCP1-dependent H+ current recorded from whole brown fat mitoplasts before (black) or after CoQ_10_ treatment (20 µM). 1 mM GDP (blue) was used as an inhibitor of UCP1 activity. Lower panel: UCP1-dependent H+ current densities in the inner mitochondrial membrane (IMM) of brown fat after CoQ_10_ treatment compared to UCP1-dependent H+ current before treatment, n = 5. No differences were observed in H+ current densities between CTL and CoQ_10-_treated samples. Mean ± SEM. (**f**) UCP1 protein expression in murine brown adipocyte cells, measured by western blot, was decreased in 4CBA-treated cells compared to CTL, n = 3. (**g**) Thermogenic genes were measured by reverse transcription polymerase chain reaction (rtPCR), n = 6. UCP1 gene expression was decreased in 4CBA-treated cells. (**h**) Stromal vascular fraction (SVF) was isolated from brown adipose tissue (BAT) of PDSS2FL animals, and PDSS2 was knockout by the induction of cre recombinase with doxycycline. Gene expression was measured by rtPCR, n = 4. PDSS2 and UCP1 gene expression was decreased while Cre expression was increased. Data are mean ± SEM. Significance presented at * *p* < 0.05, ** *p* < 0.01, and *** *p* < 0.001 compared to controls or otherwise indicated.

**Figure A1:**
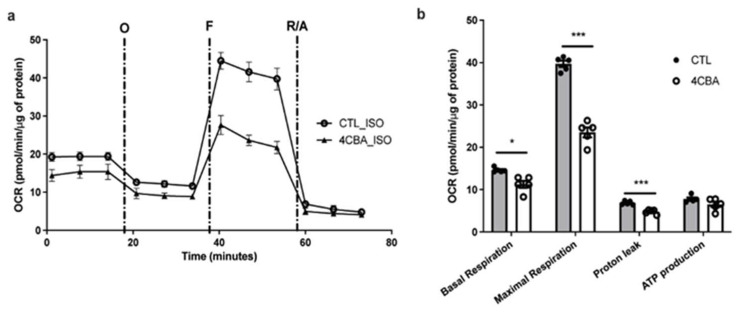
(**a**) Oxygen consumption rate (OCR) of isoproterenol and BSA-treated brown adipocyte cells were measured using SeaHorse XFe24 analyzer and components of mitochondrial respiration were analyzed, n = 5. (**b**) Basal, maximal, and proton leak OCR was decreased in 4CBA-treated cells compared to cells treated with vehicle (CTL). Data represent mean ± SEM. Significance presented at * *p* < 0.05, and *** *p* < 0.001 compared to controls or otherwise indicated.

To investigate the impact of CoQ deficiency on respiration and thermogenic function in brown adipocytes, we treated mature murine brown adipocytes (sBAT) [18] with the CoQ biosynthesis inhibitor 4-Carboxybenzaldehyde (4CBA) [23]. Total CoQ levels, dominated by CoQ_9_, were reduced by ~60% with 4 mM µM 4CBA treatment for 48 h (Figure 1a). The impact of CoQ deficiency on cellular respiration in sBAT was examined using a standard mitochondrial stress test (Figure 1b). Surprisingly, while basal, maximal, and uncoupled respiration were affected by 4CBA treatment, the most significant decrease, 50% suppression, was observed with uncoupled respiration, while ATP production was unchanged (Figure 1c). These results were reproduced under UCP1-activating conditions in the presence of 2% BSA and 2 µM isoproterenol (Figure A1a,b). Expression of major electron transport chain complexes was unaffected by CoQ deficiency based on Western blots (Figure 1d). As CoQ had been suggested to act as an obligatory cofactor for UCP1 [24], we next performed mitochondrial patch clamp experiments with and without CoQ supplementation as previously described [25]. Supplementation with CoQ_10_ did not affect the inner mitochondrial membrane proton leak suggesting that the defects in respiration we observe are not due to CoQ’s effect on UCP1 activity (Figure 1e). However, CoQ deficiency triggered a robust suppression of the UCP1 gene and protein expression and downregulated several key brown/beige transcriptional regulators, including PGC1α, EBF2, and PPARγ, but not PRDM16 (Figure 1f,g). This suggests that decreased UCP1 expression, rather than defective electron transfer or decreased UCP1 activity, dominates the observed decrease in basal and uncoupled respiration. To confirm that the downregulation of UCP1 was due to CoQ deficiency, we employed a genetic loss of function system. To this end, stromal vascular fraction (SVF) cells from BAT of decaprenyl diphosphate synthase subunit 2 (PDSS2), an obligatory coenzyme Q biosynthetic enzyme, floxed mice [16] were infected with a lentivirus containing Cre recombinase resulting in decreased PDSS2 expression. As with pharmacological CoQ deficiency, genetic suppression of CoQ synthesis in BAT cells resulted in greatly decreased UCP1 gene expression (Figure 1h).

### 3.2. CoQ Deficiency Driven UCP1 Suppression Is Specific to Brown and Beige Fat

**Figure 2:**
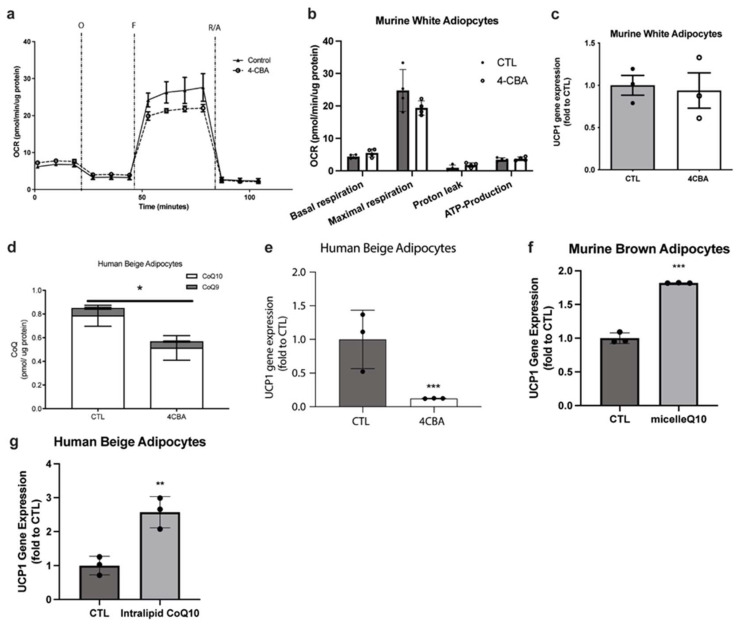
CoQ deficiency does not affect white adipocyte respiration and UCP1 levels. CoQ supplementation restores UCP1 levels in murine brown adipocytes and human beige adipocytes. (**a**,**b**) OCR of white murine adipocytes, which remained unchanged between CTL and 4CBA treated cells, were measured using a seahorse XFe24 analyzer, and components of mitochondrial respiration were analyzed, n = 4. (**c**) UCP1 gene expression, measured using rtPCR, was not significantly changed in white murine adipocytes treated with 4CBA compared to CTL. (**d**) CoQ levels were decreased in human beige adipocytes treated with 4CBA compared to CTL, n = 3. Data are mean ± SEM. (**e**) UCP1 gene expression of human beige adipocytes, measured using rtPCR, treated with 4CBA was decreased compared to CTL. (**f**) UCP1 gene expression in murine brown adipocytes treated with a micellar formulation of CoQ_10_ (n = 3) and (**g**) UCP1 gene expression in human beige adipocytes treated with an intralipid formulation of CoQ_10_ (n = 3), measured using rtPCR, was increased compared to cell treated with vehicle (CTL). Data are mean ± SEM. Significance presented at * *p* < 0.05, ** *p* < 0.01, and *** *p* < 0.001 compared to controls or otherwise indicated.

To determine the generality of the observed CoQ deficiency effects on adipocytes, we analyzed the effect of CoQ synthesis suppression on white adipocytes. Fully differentiated 3T3L1 adipocytes were treated with 4CBA as above and analyzed via a mitochondrial stress test. Interestingly, we did not observe any significant changes in oxygen consumption rates, nor the very low-level expression of UCP1 (Figure 2a–c). Conversely, in other UCP1-expressing cell types, particularly human beige adipocytes, reduction of total CoQ levels, dominated in this case by CoQ_10_, also resulted in a robust suppression of UCP1 (Figure 2d,e) indicating that this novel effect of CoQ holds true across UCP1 positive cell types and across species. Next, we determined if superphysiological levels of CoQ can further enhance UCP1 expression in brown and beige adipocytes. Due to its low solubility in water and its limited bioavailability when supplemented in its naked form, CoQ_10_ was supplemented in either a micelle or intralipid form, allowing increased absorbance efficiency into cells. Indeed, supplementation of murine brown adipocytes and human beige adipocytes with 2 µM and 10 µM exogenous CoQ_10_, respectively resulted in increased UCP1 expression (Figure 2f,g). This shows a direct relationship between CoQ levels in brown and beige adipocytes and UCP1 expression.

### 3.3. BAT CoQ Deficiency Alters Cellular Bioenergetics and Redox Functions

**Figure 3:**
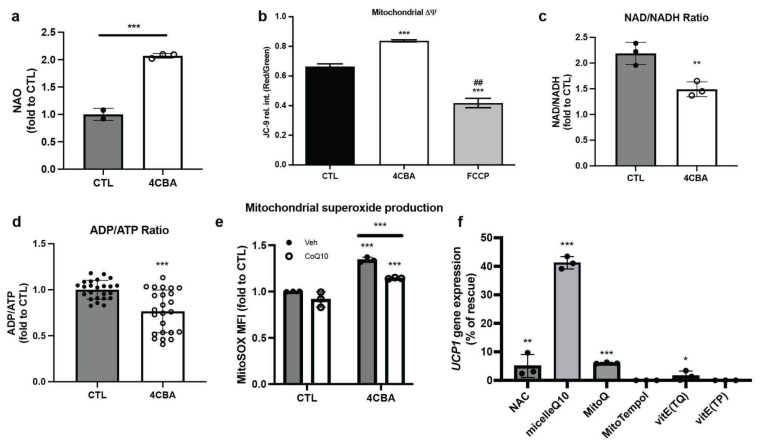
BAT CoQ deficiency alters mitochondrial bioenergetics and redox function. (**a**) Mitochondrial mass, measured by flow cytometry using nonyl acridine orange (NAO), a fluorescent dye, was increased after treatment with 4CBA in comparison to the vehicle (CTL), n = 3. (**b**) Mitochondrial membrane potential ΔΨ was detected using fluorescent dye JC-9 by confocal microscope and was found to be increased in 4CBA treated cells. Green and red fluorescence intensity was measured from 4 randomly selected images using Imaris software and presented in a Red/Green ratio. ## *p* < 0.01 compared to 4CBA treatment. (**c**) NAD+ to NADH and (**d**) ADP to ATP ratios were measured using an ELISA kit and were both decreased after treatment with 4CBA. (**e**) Mitochondrial superoxide in brown adipocytes was increased after 4CBA treatment. CoQ_10_ supplementation partially rescued superoxide production in 4CBA-treated cells. Mitochondrial superoxide production was detected using MitoSOX by flow cytometry, n = 3. 4CBA treatment (**f**) Murine brown adipocytes were treated with 4CBA in the presence of various antioxidants, n = 3. MicelleQ10 rescued UCP1 suppression by ~45%. Data represent mean ± SEM. Significance presented at * *p* < 0.05, ** *p* < 0.01, and *** *p* < 0.001 compared to controls or otherwise indicated.

To further characterize changes in BAT mitochondrial function, we stained sBAT cells with 10-N-nonyl-acridine orange chloride (NAO) [26] with and without 4CBA treatment and found, despite the decreased cellular respiration rates, an increase of mitochondrial mass (Figure 3a) was observed. This could be in part due to defective autophagy under oxidative stress and thus the accumulation of dysfunctional mitochondria [27]. Mitochondrial membrane potential (ΔΨ), measured using JC-9 [28], was increased (Figure 3b) as expected from the decreased uncoupled respiration and UCP1 expression. In line with the decreased uncoupled respiration and higher membrane potential, NAD+/NADH and ADP/ATP ratios were decreased (Figure 3c,d). High ΔΨ has been associated with increased reactive oxidative species (ROS) production [29]. Since CoQ is a known cellular antioxidant, we measured mitochondrial reactive oxygen species (ROS) production in response to CoQ deficiency. Indeed, we found increased mitochondrial superoxide production in 4CBA-treated cells measured using MitoSOX, a fluorogenic dye targeted to mitochondria for superoxide detection (Figure 3e). We then incubated cells with either the ROS inhibitors N-acetyl cysteine (NAC, at 250 µM), the mitochondrially targeted CoQ analog mitoquinone (mitoQ) [30,31] (2 µM), the mitochondria-targeted superoxide dismutase mimetic MitoTEMPOL [32] (2 µM), the vitamin E antioxidants alpha-tocopherol phosphate disodium salt (TP, 10 µM) alpha-tocopheryl quinone (TQ, 10 µM), and a micelle formulation of CoQ_10_ (2 µM). Surprisingly, most of the well-known antioxidants had only modest effects on the recovery of UCP1 expression (Figure 3f). In contrast CoQ_10_ supplementation was able to rescue over 40% of the UCP1 expression, suggesting both an involvement of ROS as well as a unique role for CoQ in suppression of UCP1 expression.

### 3.4. CoQ Deficiency Leads to BAT Dysfunction In Vivo

**Figure 4:**
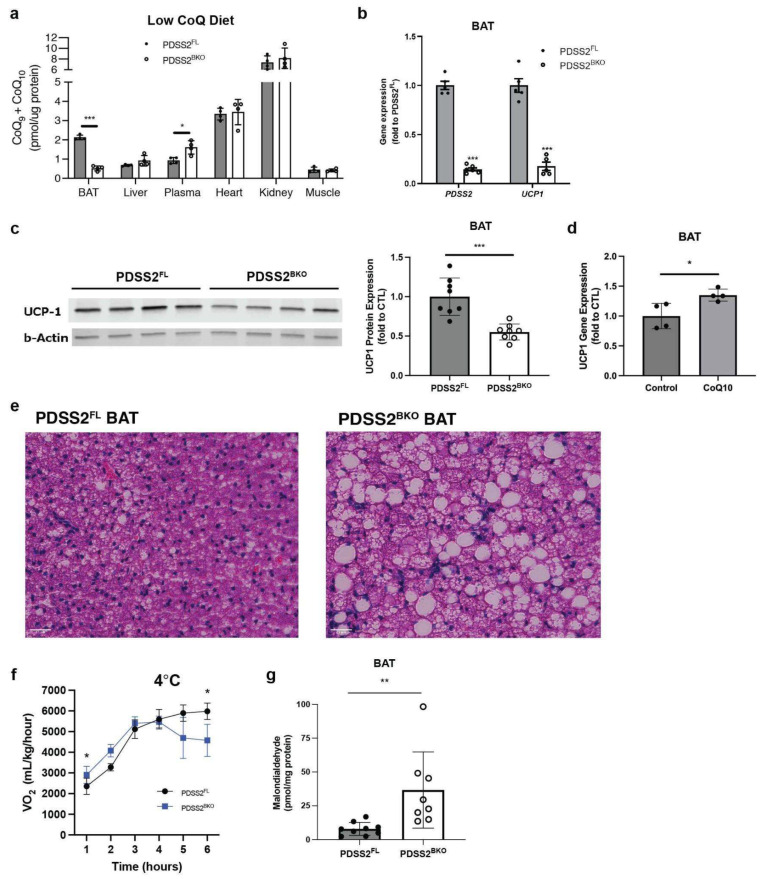
CoQ deficiency leads to BAT dysfunction in vivo. (**a**) CoQ levels in BAT of BAT-specific PDSS2 knockout mice (PDSS2^BKO^) were decreased compared to controls (PDSS2^FL^) when mice were fed a defined low CoQ diet. CoQ levels in other tissues and plasma were also measured, n = 4. (**b**) PDSS2 and UCP1 gene expression, measured by rtPCR, in BAT was decreased in PDSS2^BKO^ compared to PDSS2^FL^, n = 5–6. (**c**) UCP1 protein levels in BAT from knockout mice compared to control mice were decreased when assessed and quantified using western blot, n = 8. Each lane in the western blot pictured is one independent sample. (**d**) UCP1 gene expression was measured from BAT of wildtype mice fed a defined low CoQ diet with or without a CoQ_10_ supplement. CoQ_10_ supplementation to the diet increased UCP1 gene expression, n = 4. (**e**) Hematoxylin & Eosin (H&E) staining of BAT from PDSS2^FL^ and PDSS2^BKO^ animals, scale bar = 25 µm. BAT from PDSS2^BKO^ animals presents a whitening phenotype compared to BAT from PDSS2^FL^. (**f**) Oxygen consumption of PDSS2^FL^and PDSS2^BKO^ animals at 4 °C, measured by Comprehensive Lab Animal Monitoring System (CLAMS) (n = 3/group), showed increased cold sensitivity in PDSS2^BKO^ animals. (**g**) Lipid peroxidation products malondialdehyde (MDA), measured using a TBARS assay (KGE013, R&D), was increased in BAT from knockout mice compared to controls, n = 8–9. Data represent mean ± SEM. Significance presented at * *p* < 0.05, ** *p* < 0.01, and *** *p* < 0.001 compared to controls or otherwise indicated.

**Figure A2:**
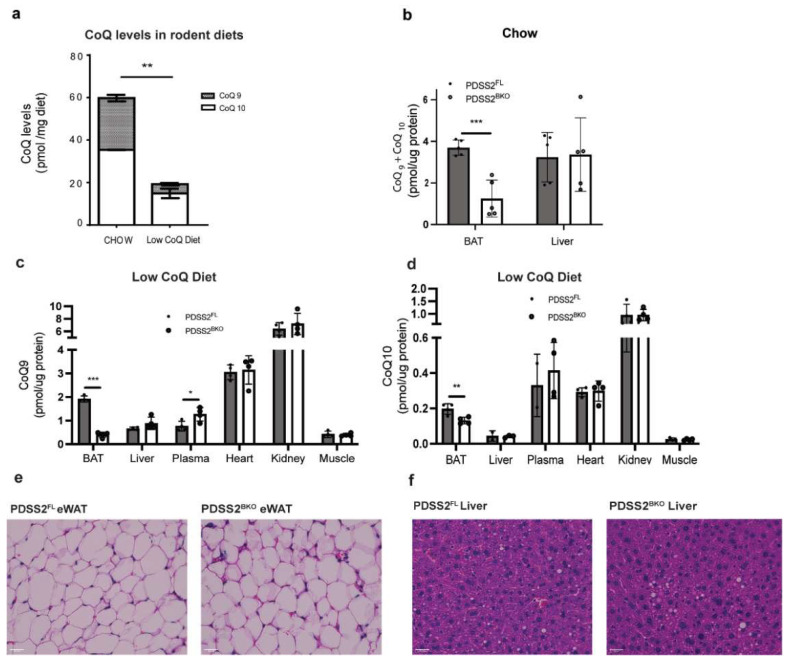
(**a**) Levels of CoQ9 and CoQ10 were lower in a low CoQ diet compared to a normal chow diet. (**b**) Combined CoQ_9_ and CoQ_10_ measurements in BAT and liver of PDSS2^BKO^ and PDSS2^FL^ mice fed a normal chow diet, n = 5. CoQ levels in BAT of PDSS2^BKO^ are decreased to 50% of PDSS2^FL^ and unchanged in the liver. (**c**,**d**) Individual CoQ_9_ and CoQ_10_ measurements in tissues of PDSS2^BKO^ and PDSS2^FL^ mice fed a defined low CoQ diet, n = 4. H&E staining of (**e**) eWAT and (**f**) liver of PDSS2^FL^ and PDSS2^BKO^ animals show no notable morphological differences. Data represent mean ± SEM. Significance presented at * *p* < 0.05, ** *p* < 0.01, and *** *p* < 0.001 compared to controls or otherwise indicated.

To examine BAT CoQ deficiency in vivo, we generated a brown/beige fat-specific knockout strain for the CoQ synthesis enzyme PDSS2. To this end, a UCP1-cre line was crossed with a PDSS2 Flox strain [16], both in the C57 background, thus generating the PDSS2^BKO^ line. PDSS2BKO animals display abrogation of PDSS2 expression (Figure 4b) in BAT. When mice are fed a normal chow diet, BAT CoQ levels in PDSS2BKO animals are reduced to 50% compared to PDSS2^FL^ (Figure A2b). However, we noticed that standard rodent chow contains high levels of CoQ9 and −10 (60 pmol/mg). To better define our model and to exclude that CoQ levels in regular chow could mask PDSS2BKO phenotype, we switched animals to a defined chow containing 32% of the CoQ content present in normal chow (Figure A2a), for two to three weeks prior to experiments. This further reduced CoQ levels in PDSS2BKO BAT to 25% compared to PDSS2FL. CoQ levels in other tissues were unaffected while plasma CoQ levels were increased (Figure 4a and Figure A2c,d). Importantly, a pronounced suppression of the UCP1 gene and protein expression was detected in BAT tissues of the PDSS2BKO animals (Figure 4b,c). Further, when wild-type C57BL6/j mice were fed a defined, low CoQ diet supplemented with exogenous CoQ_10_ for seven weeks, UCP1 gene expression in brown adipose tissue was increased (Figure 4d) similar to our in vitro findings with exogenous CoQ_10_ supplementation to cells. Hematoxylin and eosin (H&E) staining of BAT revealed that some brown adipocytes in PDSS2BKO animals have a unilocular lipid droplet morphology, suggesting a more white adipose tissue (WAT)-like phenotype (Figure 4e), in line with the observed decrease in expression of UCP1. This is not observed in BAT of PDSS2FL controls and further supports the notion that CoQ deficiency causes BAT dysfunction. Unlike BAT, epidydimal WAT (eWAT) and liver did not display apparent changes to morphology (Figure A2e,f). We then studied how these alterations in BAT morphology and UCP1 expression affect the cold tolerance of PDSS2BKO animals. Mice were fed a defined low CoQ diet for two to three weeks, then mice were placed into a Comprehensive Lab Animal Monitoring System (CLAMS) metabolic chamber in order to measure their oxygen consumption rate at 4 °C. When PDSS2BKO animals were exposed to a 4 °C cold challenge, an immediate and dramatic respiration defect became apparent with significantly lower VO_2_ rates. Mice became hypothermic after 5–6 h at 4 °C and had to be removed from the metabolic chamber due to animal welfare concerns (Figure 4f). Lastly, because of CoQ’s ability to act as an antioxidant, lipid peroxidation was measured in the BAT of our mouse model. We found that levels of malondialdehyde were significantly upregulated in BAT from our PDSS2BKO animals suggesting more oxidative damage in CoQ-deficient BAT (Figure 4g). Overall, we show that our CoQ deficiency phenotype can be replicated in vivo and that suppression of UCP1 expression in BAT leads to changes in BAT morphology and impaired cold tolerance in CoQ-deficient animals.

## 4. Discussion

In this study, we established that CoQ deficiency in brown adipose tissue (BAT) results in impaired oxygen consumption, notably a 50% reduction in uncoupled respiration and a 40% reduction in basal respiration (Figure 1b), resulting from uncoupling protein 1 (UCP) expression suppression (Figure 1f). UCP1 expression suppression could be replicated in human beige adipocytes (Figure 2e) but not in murine white adipocytes (Figure 2c) suggesting that major effects on cellular respiration of CoQ deficiency are driven by UCP1 suppression and are thus specific to brown and beige fat. BAT CoQ deficiency also resulted in changes to cellular bioenergetics, including increased membrane potential (Figure 3b), decreased ADP/ATP ratios (Figure 3d), and increased mitochondrial reactive oxygen species (ROS) production (Figure 3e) alongside increased mitochondrial mass (Figure 3a), reflecting a possible impairment in the complex mitochondrial quality control machinery leading to an alteration of mitophagy events [27]. By creating a UCP1-cre driven decaprenyl diphosphate synthase subunit 2 (PDSS2) conditional knockout strain, we were able to produce a BAT-specific CoQ deficiency in vivo model (75% reduction in total BAT CoQ) (Figure 4b). As with the in vitro systems, UCP1 expression was decreased in BAT subsequent to CoQ deficiency (Figure 4a), resulting in impaired cold tolerance and morphological alterations to BAT in PDSS2^BKO^ animals (Figure 4e–g). Exogenous CoQ supplementation, both in vitro (Figure 2f,g) and in vivo (Figure 4d) resulted in increased UCP1 expression providing a positive correlation between CoQ levels and UCP1 expression in BAT.

The PDSS2^BKO^ line provides a first insight into the brown adipose tissue-specific effects of CoQ deficiency. Although primary CoQ deficiencies (MIM 607426) are rare and manifest from mutations in CoQ biosynthesis genes, secondary CoQ deficiency encompasses a broader spectrum of disease and is also associated with drug treatments such as the use of 3-hydroxy-3-methylglutaryl coenzyme-A (HMG-CoA) reductase inhibitors (frequently referred to as statins), which is widely used for lowering cholesterol. The effects of CoQ deficiency on specific tissues are still poorly understood but are thought to disproportionately affect cells with high energy demands, such as BAT. BAT and, to a lesser extent, beige adipose tissue, are present in all mammals, including humans [33], and exclusively express UCP1. BAT activation and prevalence are associated with improved metabolic health, such as a lower incidence of type two diabetes mellitus [34], suggesting that CoQ deficiency-induced BAT dysfunction could result in adverse effects on patients. Indeed, studies have revealed that statin treatment is associated with decreased BAT activity [35], increased body weight, and increased risk for the development of type two diabetes [36]. Although it remains unclear if the mechanism resulting in these adverse effects of statin treatment involves CoQ deficiency, it has been reported that statin treatment in patients results in lower levels of serum CoQ levels [37], providing a potential link between statins’ adverse metabolic effects and CoQ deficiency. Even though it is difficult to make a direct comparison between our model, which focuses on BAT-specific CoQ deficiency in cells and mice, and the heterogenous clinical phenotypes of CoQ deficient patients, some commonalities were noted. Similar to our findings, decreased oxidative phosphorylation (OXPHOS) respiration and ROS production are also common aspects of several diseases linked to CoQ deficiency [38], showing the relevance of our study to patient phenotypes. Further in our in-vivo model, we found that CoQ deficiency shows a higher cold sensitivity (Figure 4f). Interestingly, in line with our finding, Rahman et al. [15] described a neonatal case of systemic CoQ10 deficiency presenting hypothermia at 6 h of age, which could be attributed to decreased UCP1 affected by CoQ lower levels. Despite this, we cannot exclude that in a systemic CoQ deficient case such as the one described by Rahman et al., other failures of cellular energy metabolism occurred.

CoQ functions extended substantially beyond the classical role as electron carriers in the respiratory chains. Among the functions described so far, the relation between CoQ and UCP is still poorly understood. In 2000 Echtay et al. reported that CoQ was an obligatory cofactor for proton transport by UCP1 [24]. Conversely, a report in 2004 [39] suggested that CoQ was not required for UCP1 proton conductance in a yeast system. This conclusion is supported by our patch-clamp analysis showing that the uncoupling function of UCP1 was independent of CoQ. Instead, CoQ deficiency causes changes in UCP1 abundance via the regulation of mRNA and protein levels. However, it remains to be elucidated how CoQ regulates the expression of UCP1 and if this occurs through direct or indirect mechanisms. Interestingly, Tiefenbach et al. showed that CoQ_10_, and its more soluble analogue, idebenone, acts as partial agonists for both peroxisome proliferator-activated receptors α (PPARα) and γ (PPARγ) in a Zebra Fish model [40]. Since UCP1 gene expression is controlled by PPARs [41], this could provide one plausible mechanistic connection.

Another interesting finding in our model is CoQ’s ability to stimulate UCP1 expression when it is exogenously supplemented both in vitro and in vivo. While enhanced UCP1 expression could result in metabolic benefits in patients, the efficacy and the benefits of CoQ supplementation remain controversial. While some studies reported improved glycemic control in diabetic patients [42,43] following CoQ supplementation, others failed to find such effects on plasma glucose or Hemoglobin A1C (HbA1c) levels [44,45]. These discrepancies could be potentially due to the low number of clinical trials and a limited number of patients [46] as well as the high variability of various CoQ formulations, treatment length, dosage, and intra-individual variations such as concomitant medications, diet, onset, and severity of the diseases. CoQ absorption and transport throughout are also poorly understood [47]. Here, we used intralipid loaded CoQ_10_ or micellar CoQ_10_ to improve absorption into cells as well as a relatively long 7-week CoQ supplementation period to study in vivo effects of CoQ on UCP1 expression. These approaches may not directly be translated to the treatment of patients, but several efforts have been carried out to find new CoQ formulations, in order to enhance its absorption through incorporation in different carriers such as liposomes, micelles, oleogels, β-cyclodextrin inclusion complexes or nanoparticles [48]. For instance, in a recent study, Hekimi et al. reported promising data regarding the effects of caspofungin analogues on CoQ_10_ solubilization and uptake [49].

Overall, the new insights into the relationship between CoQ and BAT function reported here should have significant implications for our understanding of how ubiquinone levels affect metabolic health and may provide new venues for the treatment of diseases such as type two diabetes.

## Data Availability

The data that supports the findings of this manuscript can be found in the submission or obtained from the corresponding author upon request.

## Appendix C

**Table A1:** rt-PCR primer sequences.

Gene	Exons	Forward	Reverse
UCP1	2-3	CACACCTCCAGTCATTAAGCC	CAAATCAGCTTTGCCTCACTC
PGC1a	2-3	CTCCATCTGTCAGTGCATCA	CCAACCAGTACAACAATGAGC
PRDM16	12-13	CACTTGAACGGCTTCTCTTTG	CACAAGACATCTGAGGACACA
EBF2	14-15	GGAGGTGCTGTAATTAGATTGCT	GTCAGCATTTCAGAGTCCACA
PPARG	4-5	TGCAGGTTCTACTTTGATCGC	CTGCTCCACACTATGAAGACAT
PPIA	4-5	TTCACCTTCCCAAAGACCAC	CAAACACAAACGGTTCCCAG
PDSS2	6-8	CACCTTTGCCAGCTTTGATTG	GTCTTACATCAGGAGTTTCTTGGA
Cre	N/A	CATCGCTCGACCAGTTTAGTT	CTGACGGTGGGAGAATGTTAAT

## Appendix D

**Table A2:** Antibodies.

Antibody	Source	Identifier
beta Tubulin	Abcam	Cat# ab131205, RRID:AB_11156121
beta Actin	Sigma-Aldrich	Cat# A1978, RRID:AB_476692
UCP1a	Abcam	Cat# ab209483 RRID:AB_2722676
UCP1 (E9Z2V) XP Rabbit mAb	Cell Signaling Technology	Cat# 72298
Total OXPHOS Rodent WB Antibody Cocktail	Abcam	Cat# ab110413
IRDye 680LT Goat anti-Mouse IgG antibody	LI-COR Biosciences	Cat# 925-68020, RRID:AB_2687826
IRDye 800CW Goat anti-Rabbit IgG antibody	LI-COR Biosciences	Cat# 925-32211, RRID:AB_2651127
